# Mitochondrial Metabolism in Melanoma

**DOI:** 10.3390/cells10113197

**Published:** 2021-11-16

**Authors:** Christina Huang, Rakan H. Radi, Jack L. Arbiser

**Affiliations:** 1 Department of Dermatology, School of Medicine, Emory University, Atlanta, GA 30322, USA; ckh5420@psu.edu (C.H.); rakan.h.radi@emory.edu (R.H.R.); 2 Atlanta Veterans Administration Medical Center, Decatur, GA 30033, USA; 3 Winship Cancer Institute, Emory University, Atlanta, GA 30322, USA

**Keywords:** melanoma, mitochondria, SOX2, SIRT3, MCL1

## Abstract

Melanoma and its associated alterations in cellular pathways have been growing areas of interest in research, especially as specific biological pathways are being elucidated. Some of these alterations include changes in the mitochondrial metabolism in melanoma. Many mitochondrial metabolic changes lead to differences in the survivability of cancer cells and confer resistance to targeted therapies. While extensive work has gone into characterizing mechanisms of resistance, the role of mitochondrial adaptation as a mode of resistance is not completely understood. In this review, we wish to explore mitochondrial metabolism in melanoma and how it impacts modes of resistance. There are several genes that play a major role in melanoma mitochondrial metabolism which require a full understanding to optimally target melanoma. These include BRAF, CRAF, SOX2, MCL1, TRAP1, RHOA, SRF, SIRT3, PTEN, and AKT1. We will be discussing the role of these genes in melanoma in greater detail. An enhanced understanding of mitochondrial metabolism and these modes of resistance may result in novel combinatorial and sequential therapies that may lead to greater therapeutic benefit.

## 1. Introduction

Melanoma is a common solid tumor which is capable of rapid growth, long periods of dormancy, late recurrence, and widespread metastases [1,2,3,4,5]. It is highly likely that all these states of melanoma represent a differing metabolism [5]. It has long been recognized that tumor cells, including melanoma, have a different metabolic profile to normal cells [6]. Most normal cells use respiration as their main mode of ATP generation, with some exceptions including certain cells in brain tissue which have been observed to employ glycolysis on occasion [6,7]. Conversely, it has long been held that cancer cells often appear to use a glycolytic metabolism [8]. The initial recognition of this phenomenon was made by Otto Warburg, who observed that tumor cells often undergo glycolysis even in the presence of sufficient oxygen, a process known as aerobic glycolysis [9]. Although glycolysis can be an important driver for cancerous growth, more recent studies have demonstrated that some cancers, such as melanoma, are more versatile in their metabolism, allowing adaptation for a variety of factors [10].

Rapid cancerous growth poses unique challenges in metabolism not seen in normal tissues, such as hypoxia (as cellular demand for oxygen exceeds supply, and cells grow away from available vasculature), altered microenvironment, lack of differentiation, and acidosis. Therefore, melanoma cells can shuttle between glycolysis and respiration depending upon conditions of growth, hypoxia, acidosis, chemotherapy, and radiation [11,12,13,14]. This phenomenon is known as plasticity. Cancer cells are plastic and tumors are heterogenous, containing cells which are both glycolytic and respiratory. In melanoma, cells can shift towards respiration to meet certain energetic demands. For instance, melanoma cells display metabolic diversity as up to 35–50% of wild-type, BRAF-mutant, and patient-derived cells display high oxidative phosphorylation activity, employing a respiratory metabolic strategy to meet energy needs [12]. This could be favored in a state of glucose deprivation, where cells have insufficient substrate for glycolysis.

Meanwhile, cells in the center of a tumor, which are deprived of oxygen as they are farther from the blood supply, may be more dependent on glycolysis [15,16]. Glycolysis might also confer survival advantages under conditions of hypoxia, a state that reduces the efficacy of radiation therapy, immunotherapy, and chemotherapy [12,17]. Even under aerobic conditions, glycolysis may possibly confer a survival advantage by activating the nuclear factor kappa B (NF-κB) protein complex, subsequently promoting resistance to radiation and chemotherapy [18]. Plasticity also allows for adaptation to the tumor microenvironment. For instance, a more acidic microenvironment (from lactic acid, a by-product of glycolysis and subsequent fermentation) induces a shift towards oxidative phosphorylation in cells [19,20]. Melanoma cells in vitro have demonstrated both elevated respiratory and glycolytic activity compared to melanocytes, showing use of both metabolic strategies [15].

Importantly, metabolic plasticity has posed a challenge when it comes to treatment. Targeted therapies have revolutionized the practice of oncology, both for hematopoietic malignancies and solid tumors. Most agents in current clinical use target a kinase and/or driver mutation that is constitutively active in a malignancy [21,22]. The discovery of the BCR-ABL1 translocation as the driving event in chronic myelogenous leukemia was the initial event that catalyzed the development of targeted therapies against driver oncogenes [23]. Since that initial discovery, targeted therapies have been developed for the treatment of melanoma, such as BRAF and MAP2K1/2 inhibitor drugs [24]. While impressive responses and prolonged survival are observed, it has become clear that these therapies are not completely curative as resistance may develop. This has led to the extensive study of mechanisms of resistance to targeted therapies [25]. Despite the plethora of mechanisms of resistance, including the activation of other oncogenes and downregulations of tumor suppressors, many pathways converge onto mitochondrial responses. For instance, BRAF inhibitor-resistant melanoma cells are observed to have elevated reactive oxygen species (ROS) and correspondingly display higher levels of the reactive oxygen detoxification enzyme SOD2 [26].

Multiple mitochondrial responses and metabolic shifts facilitate resistance to targeted treatments [5]. Understanding specific factors and processes implicated in metabolic shifts could help develop ways to restrict plasticity or target specific changes seen with plasticity. For example, tumor cells undergo certain adaptations to conduct respiration. These include the use of non-glucose substrates in respiration (glutamate, fatty acid oxidation) and mitochondrial adaptations which preserve mitochondrial respiration, such as favoring mitochondrial fusion over fission [27,28,29,30]. One such major mitochondrial adaptation is the overexpression of the MCL1 apoptosis regulator gene (MCL1), preventing apoptosis in melanoma cells and driving mitochondrial fusion to promote respiration [31]. Another major mediator of mitochondrial responses is the mitochondrial deacetylate sirtuin 3 (SIRT3) which stimulates mitochondrial biogenesis and cellular respiration [32]. The pharmacologic manipulation of factors influencing metabolism such as MCL1 and SIRT3 may enhance the response of tumors to targeted therapies [33]. Other key metabolic factors affecting mitochondrial metabolism in melanoma include BRAF, CRAF, SOX2, TRAP1, RHOA, SRF, PTEN, and AKT1, which play varying roles in modulating bioenergetics and mitochondrial responses. The factors that will be discussed, along with their respective functions and roles in metabolic plasticity, are summarized in Table 1.

## 2. BRAF—A Major Driver towards Glycolysis

BRAF is a key kinase in the mitogen-activated protein kinase (MAPK) signaling pathway which is essential for many functions including cellular proliferation and survival [56]. It is regulated by upstream epidermal growth factor receptor (EGFR) and members of the RAS type GTPase family. In turn, BRAF activates downstream MAP2K (also referred to as MEK) which then activates MAPK (also referred to as ERK) [57]. BRAF mutations are the most common driver mutation in melanoma, accounting for approximately 50% of cases, and among these, 90% of mutations are BRAF^V600E^ [57]. Metabolically, BRAF activity suppresses oxidative phosphorylation and drives glycolysis. This shift to glycolysis also helps prevent oncogene-induced senescence, avoiding reactive oxygen species (ROS) produced during respiration [49]. In addition, this aerobic glycolysis inhibits effectiveness of immunotherapy by creating an acidic tumor microenvironment [58].

Upregulated BRAF activity increases aerobic glycolysis through the activation of hypoxia inducible factor 1 subunit alpha (HIF1A), a key regulator for glycolysis [5]. HIF1A is usually activated in conditions of hypoxia, promoting a shift towards anaerobic glycolysis to meet bioenergetic needs when oxidative phosphorylation is not possible [59]. Through HIF1A activity, pyruvate dehydrogenase is inactivated, preventing pyruvate being converted into acetyl-CoA and subsequently inhibiting citric acid cycle activity [59]. Microarray experiments have shown that melanoma cell lines with BRAF^V600E^ displayed increased HIF1A expression [60]. Furthermore, the expression of HIF1A is maintained even without hypoxia in melanoma cells, and one study found that the expression of HIF1A mRNA and protein was increased the most in metastatic melanoma colonies, followed by vertical growth phase, horizontal growth phase, and finally normal melanocytes with the lowest expression [5,61]. Another study found that HIF1A contributes to melanoma invasion and metastasis via the activation of SRC, an oncogene, while the inactivation of HIF1A reduced metastasis [62]. This indicates the importance of a HIF1A-induced glycolytic metabolism in melanoma progression and metastasis, and a possible correlation between a glycolytic metabolism and tumor invasion and metastasis. Furthermore, through BRAF/MAPK signaling, MYC (which increases glucose uptake and hexokinase activity) is upregulated, further driving glycolysis, while also promoting glutamine usage [5,63].

**Figure 1:**
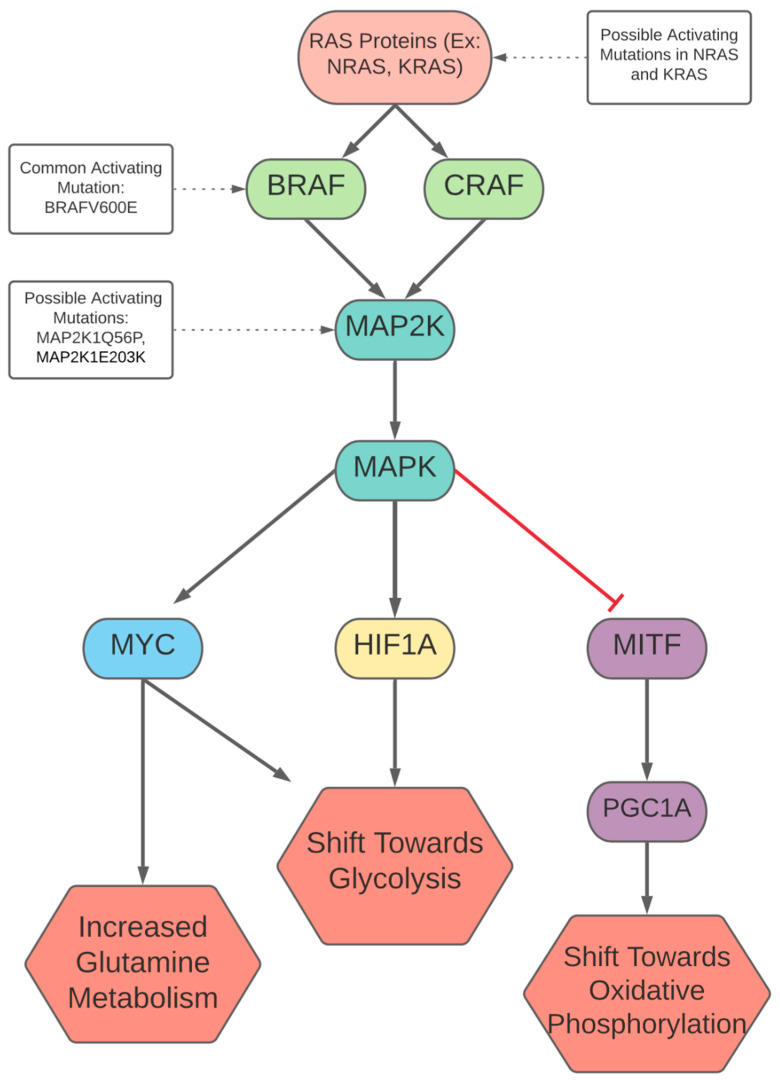
Signaling pathways involving BRAF and CRAF and effects on metabolism.

Aside from activating genes associated with glycolysis, BRAF/MAPK signaling also plays a role in modulating oxidative phosphorylation via melanocyte-inducing transcription factor (MITF). MITF is closely associated with plasticity in melanoma, and reportedly regulates PPARGC1A (also known as PGC1A), which in turn has a net effect of shifting metabolism towards oxidative phosphorylation [34,64]. PGC1A stimulation results in mitochondrial elongation and increased respiratory gene expression [34,65]. PGC1A also contributes to this shift by stimulating mitochondrial biogenesis (thereby increasing capacity for cellular respiration) and inducing factors such as SIRT3 that contribute to a metabolic shift towards oxidative phosphorylation [66]. Furthermore, cells with increased cellular respiration displayed reduced lactate, glucose, and glutamine dependence, with metabolic substrates being diverted to the citric acid cycle. [67]. Beyond increasing oxidative phosphorylation, PGC1A also increases ROS detoxification, allowing greater resistance to oxidative stress and metastatic potential [5,68]. In melanoma, MITF has been observed to be both up and downregulated, and the downregulation of MITF is associated with a more metastatic and treatment-resistant phenotype, suggesting an MITF-driven oxidative phenotype might be easier to treat in certain states of cancer progression [69]. The BRAF/MAPK signaling pathway increases MITF activation, but also, seemingly paradoxically, has been reported to contribute to MITF degradation. In terms of the net effect, a study found that high MAPK activation was correlated with decreased levels of MITF, while the inhibition of MAPK signaling led to an increase in MITF-mediated PGC1A induction [34]. This seems to indicate the BRAF expression suppresses MITF expression and, consequently, oxidative phosphorylation. However, MITF signaling may be more complex beyond modulating a shift towards oxidative phosphorylation, as another study found MITF targets HIF1A via the alpha-melanocyte stimulating hormone/cyclic AMP pathway [5,70]. This highlights how some mechanisms of metabolic plasticity are not as straightforward, and an improved understanding is needed to better appreciate how certain conditions favor certain pathways and metabolic strategies. The BRAF/MAPK signaling pathway and metabolic effects are summarized in Figure 1.

The discovery of BRAF overactivation as a common denominator in most melanomas led to the development of BRAF inhibitors (vemurafenib and dabrafenib). BRAF inhibitors were introduced to the clinic, resulting in impressive clinical responses. For example, in a phase III clinical trial, vemurafenib increased 12-month survival to 55% in patients with advanced melanoma compared to 43% in patients treated with the chemotherapeutic agent dacarbazine [36]. Unfortunately, responses were not durable in a significant number of cases: after 18 months of treatment with vemurafenib, only approximately 14% of patients experienced durable drug response and no relapse, as melanoma cells developed resistance to BRAF inhibition [35]. BRAF inhibitor resistance in melanoma seems remarkably adaptable, as there are so many diverse mutations that have been reported [71]. This may be consistent with the relatively high mutational burden in melanoma, and selection under conditions of BRAF inhibition may cause DNA repair stress, leading to additional driver mutations [72]. Mechanisms of resistance to BRAF inhibitors have been extensively studied. These include NRAS^Q61^ mutations, or mutations in the downstream MAP2K1 gene (MAP2K1^Q56P^ or MAP2K1^E203K^) [73,74]. These mutations activate effectors such as CRAF that have the same downstream targets of BRAF, or directly activate downstream targets of BRAF. Of interest, baseline mutations in MAP2K1^P124^ with BRAF^V600E^ were present in 7/92 samples in one study, indicating pre-existing mechanisms of intrinsic resistance [73]. Other driver mutations commonly observed in resistance to combined BRAF inhibition include KRAS, PIK3CA, AKT1, and AKT3 [25,75]. The increased expression of additional genes including MET and YAP1 signaling appears to underlie additional mechanisms of BRAF inhibitor resistance [76,77,78,79,80,81].

A common mechanism is that all forms of resistance to BRAF inhibitors result in the re-expression of MAPK, which in turn drives the glycolytic metabolic changes associated with the activation of the pathway [82]. Treatments have addressed these BRAF inhibitor resistances, such as using MAP2K1 inhibition against mutations that re-activate MAPK1/3 signaling. These treatments showed improvements in patient survival, despite adaptations that confer double resistance to this combined therapy [75,83]. Mechanisms of double resistance include the compensatory upregulation of MAPK7, a regulator of mitochondrial transcription [84]. While the initial response is enhanced by double blockade, resistance occurs as well and these resistance patterns are mediated in part by mitochondrial metabolism. For instance, BRAF inhibition results in the higher expression of PGC1A, allowing cells to maintain a bioenergetic threshold through oxidative phosphorylation [67].

Disrupting compensatory oxidative phosphorylation that occurs in response to MAPK pathway inhibitor resistance has been shown to be effective in reducing tumor growth in certain models. Metformin, a drug commonly used to treat Type II diabetes, inhibits Complex I of the electron transport chain and thereby reduces energy from oxidative phosphorylation [85]. Metformin-induced disruption of ATP production via inhibition of Complex I and the subsequent upregulation of AMPK has a variety of effects affecting metabolism including the inhibition of HIF1A, protein synthesis, and fatty acid synthesis [86]. Metformin also inhibits insulin-like growth factor 1, leading to the inhibition of the PI3K/AKT1 pathway which further decreases bioenergetic efficiency. When metformin was combined with the MAP2K1/2 inhibitor binimetinib, the combination treatment showed a synergistic effect in reducing melanoma colony formation and the growth of tumor spheroids [87]. This mechanism of elevated respiration for resistance through PGC1A activation could also be countered by downregulating MITF-PGC1A signaling, as a study that excluded MITF from the nucleus using mTORC1/2 inhibition combined with MAP2K1/2 inhibition observed apoptosis of BRAF^V600E^ cells displaying high respiration [88].

In glycolytic BRAF^V600E^ cells, the shift towards glycolysis was also accompanied by an increase in glutaminolysis as glutamine is used as an alternative to replenish citric acid cycle substrates with glycolysis decoupling from the citric acid cycle [89]. With both enhanced glycolysis and glutaminolysis to meet energy and biosynthetic demands, targeting autophagy, which is a source of glutamine for mutated cells, could disrupt cellular homeostasis [90,91]. Furthermore, mutations in the citric acid cycle enzymes isocitrate dehydrogenases 1 and 2 could confer a growth advantage in melanoma cells with mutant BRAF [5]. Examining the mutations of enzymes involved in major metabolic pathways could be another area for future research. In addition, ketogenesis associated with high dietary fat has been shown to increase acetoacetate levels, which has been implicated in driving BRAF^V600E^ tumor growth [92]. BRAF^V600E^ expression increases 3-hydroxy-3-methylglutaryl-CoA lyase (HMGCL) activity which in turn increases intracellular acetoacetate levels [93]. Acetoacetate then plays a role in enhancing binding between BRAF^V600E^ (but not wild-type BRAF) and MAP2K1, strengthening the signaling pathway [92]. This showed one facet of metabolic rewiring where the BRAF oncogene altered metabolite flux, in this case increasing acetoacetate, to drive tumor growth [93]. Hypolipidemic agents and dehydroacetic acid, an analog of acetoacetate, can, respectively, reduce levels of acetoacetate and compete with acetoacetate–BRAF association to counter this signaling enhancement effect [92]. Finally, the inhibition of the MAPK pathway in some cases also resulted in metabolic adaptation via an increase in fatty acid oxidation to compensate for metabolic stress, marking fatty acid oxidation as another resistance pathway and potential target for therapeutics [94]. Understanding the metabolic shifts that occur upon with hyperactive BRAF and subsequent inhibition could facilitate development of treatments.

### RAF1 (CRAF)-Bypassing BRAF

CRAF, also a member of the Raf protein family, can activate the same downstream effectors as BRAF [35]. CRAF overexpression appears to be a common event in BRAF inhibitor resistance but is not commonly mutated in cancers [35]. For example, altered NRAS results in the activation of CRAF as a mechanism of resistance to BRAF inhibition, as signaling is continued through CRAF signaling in a parallel path to BRAF (as shown in Figure 1), maintaining the activation of BRAF’s downstream targets [95]. Normally, CRAF is inhibited by cAMP and protein kinase A, however, mutations in RAS disrupt this inhibition, activating CRAF [96]. In cases where BRAF is inhibited, CRAF can activate downstream MAP2K1 with RAS mutation in melanoma [97]. It is likely that MAPK1/3 activation in the presence of CRAF allows tumor cells to maintain metabolism and growth in certain cell line mutations, allowing resistance to BRAF inhibition [98]. CRAF can also dimerize with BRAF, leading to increased signaling activity and resistance to treatment [99].

Beyond its ability to activate the same effectors as BRAF, CRAF and its control of mitochondrial plasticity play a role in resistance to treatment. BRAF and CRAF have homology, but also have disparate effects on mitochondrial metabolism, as a comparison of ARAF, BRAF, and CRAF demonstrated that only CRAF localizes to the mitochondria [100]. CRAF activation also caused distinct mitochondrial changes from a fused mitochondrial morphology to a fragmented and perinuclear localization that is seen with the transition from respiratory to glycolytic metabolism. The effect of CRAF is mediated by MAP2K1, as the pharmacologic inhibition of MAP2K1 with anthrax lethal toxin reverses the effect of CRAF on mitochondria [100]. Furthermore, CRAF also has anti-apoptotic effects targeting mitochondria by directly associating with apoptosis regulator BCL2 on the outer mitochondrial membrane independent of MAP2K1 signaling [101].

The CRAF/MAP2K1 interaction leading to the same downstream effects as BRAF activation can be manipulated via pharmaceutical means. One mechanism is to downregulate active CRAF expression using activators of SIRT3 (including small molecule inducers such as honokiol and hexafluoro) [47,102]. The ability of CRAF to induce the same metabolic changes seen with BRAF over-expression, even after BRAF inhibition, along with its unique role in influencing mitochondria morphology, makes CRAF an important target for research on melanoma metabolism.

## 3. SOX2—Mediating a Shift towards Oxidative Phosphorylation

**Figure 2:**
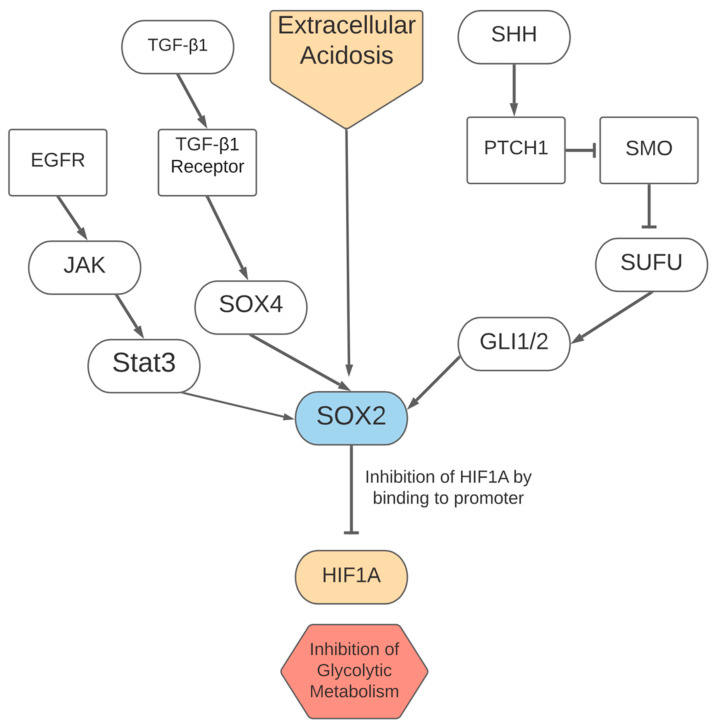
Pathways regulating SOX2 and subsequent effects of upregulated SOX2 activity on metabolism.

The expression of SRY-box transcription factor 2 (SOX2) is altered in multiple types of human malignancies, where increases in SOX2 via different pathways can result in metastasis, drug resistance, and the proliferation of cancer cells [103]. SOX2 has been observed to be amplified through a variety of pathways, including through EGFR-STAT3 signaling, TGF-β1 stimulation (mediated by SOX4), and sonic hedgehog (SHH) signaling via GLI1/GLI2 [104,105,106]. An acidic microenvironment, created by lactate as a byproduct of glycolysis and fermentation, also induces SOX2 expression as shown in Figure 2. Subsequently, SOX2 expression induces a shift towards oxidative phosphorylation, potentially to reduce glycolytic activity and prevent excessive acidity [20]. Tumors in an acidic microenvironment are significant, as they have been observed to have an aggressive phenotype. This has been associated with a relatively high use of oxidative phosphorylation and potential for cell motility, so SOX2 as a mechanism for this induction of oxidative phosphorylation would be of therapeutic interest [19,20].

SOX2 contributes to metabolic plasticity and a shift towards an oxidative metabolism in melanoma cells through multiple mechanisms. Decreasing SOX2 expression in melanoma cells was associated with a shift towards glycolysis, with increased lactate production and higher expression of SLC2A1/3 (also known as GLUT1/3), and hexokinase 2, which help import glucose and catalyze glycolysis in cells [20]. In addition, a study found that the expression of proteins important for oxidative phosphorylation, such as cytochrome C and ATP Synthase F1 Subunit Alpha were reduced with the silencing of SOX2 in acidosis-exposed melanoma, providing more evidence for the gene’s importance in the shift to oxidative metabolism [20]. The same study found, through chromatin immunoprecipitation and luciferase assays, that SOX2 directly bound the HIF1A promoter and decreased the activity of HIF1A with a dose-dependent effect, suggesting a mechanism for the shift away from glycolysis driven by SOX2 [20]. Overall, this shift towards oxidative phosphorylation induced by SOX2 embodies metabolic plasticity as it allows cells to adapt to an acidic microenvironment and might provide another mechanism for a shift to respiration under certain conditions.

Despite observations on the metabolic effects of SOX2 activation, the role of SOX2 in the initiation and progression of melanoma is somewhat unclear, as different studies yielded different conclusions regarding SOX2. One study found that higher levels of SOX2 in melanoma have been correlated with increased primary tumor thickness and invasiveness. For instance, the knockdown of SOX2 expression in A2058 melanoma cells led to a 4.5-fold decrease in invasiveness, while the overexpression of SOX2 in G361 cells via transduction was associated with a 3.8-fold increase in invasiveness in vitro [107]. However, other evidence suggests that the inactivation of SOX2 has no effect on melanoma initiation or development in vivo, where xenotransplanted SOX2 negative M050829 melanoma cells (created through CRISPR-Cas9 editing) and control M050829 cells produced similar growth patterns in mice [108]. Other evidence showed that SOX2 knockdown in A2058 melanoma cells led to similar growth patterns to control cells in vitro, but SOX2 knockdown in vivo resulted in decreased tumor growth [109]. Granted, these contrasting results could arise from the differences in cell lines and models employed [110]. In addition, because of the nature of plasticity, different microenvironments or stages of tumor development could lead to differences in whether cells favor growth driven by glycolysis or oxidative phosphorylation [41]. These seemingly contradictory results help show the nuance of metabolic plasticity.

Overall, SOX2 plays a key role in inhibiting HIF1A and inducing oxidative phosphorylation and enabling melanoma cells to shift metabolism in response to an acidic microenvironment. This has made it an interesting target for potentially limiting metabolic plasticity in tumor cells. Granted, there have been challenges in directly targeting SOX2 given its nature as a transcription factor. However, other ways to inhibit the effects of SOX2, such as targeting its upstream activators (for example, EGFR can be inhibited by gefitnib), are being examined [102].

## 4. MCL1—Maintaining Integrity of Mitochondria in Melanoma Cells

Overexpression of MCL1 is a distinctive mitochondrial adaptation found in respiratory tumorigenesis, a state of tumor growth with preserved cellular respiration [31]. MCL1 is an anti-apoptotic factor and its presence is a negative prognostic marker in many malignancies [111,112]. High levels of MCL1 protein are present in respiring tumor cells but not in normal, non-cancerous respiring cells [31]. Of interest, MCL1 has two isoforms, with one found in the outer mitochondrial membrane and the other found in the mitochondrial matrix. The outer membrane isoform of MCL1 helps keep mitochondria intact by opposing BAX and BAK, proteins that permeabilize mitochondria if activated, preventing apoptosis [31,113]. The other isoform is an amino terminal-truncated protein which localizes to the mitochondrial matrix, regulating mitochondrial fusion and promoting ATP production. The matrix isoform protects the integrity of the structure of the inner mitochondrial membrane, corrects delays in fusion present in cells with MCL1 deletion, and contributes to a stronger proton gradient in oxidative phosphorylation to maintain ATP production [31]. Overall, this indicates that the matrix isoform of MCL1 could play a role in enhancing oxidative phosphorylation in the metabolism of melanoma cells.

**Figure 3:**
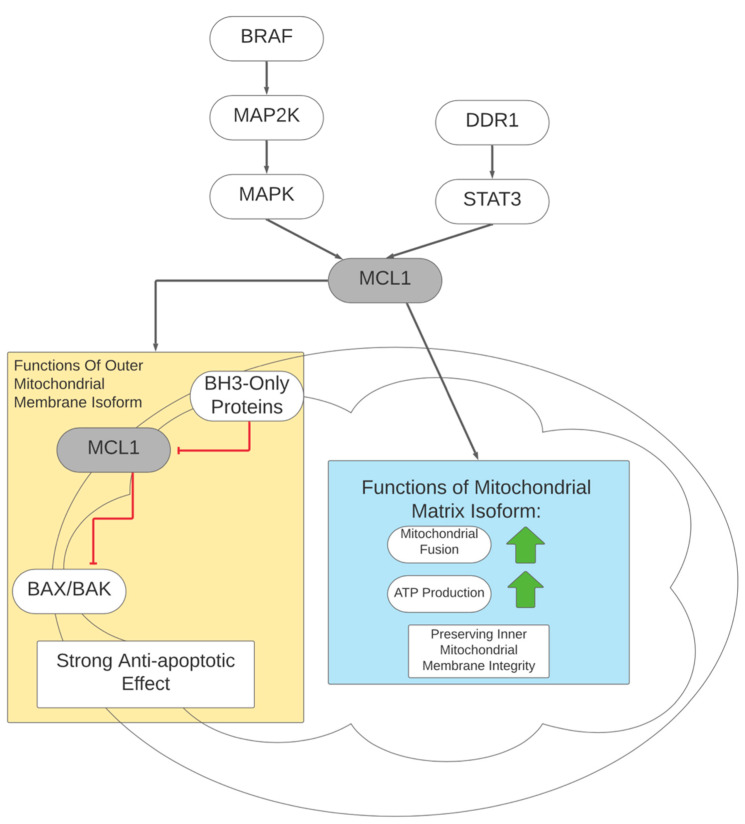
Regulation of MCL1 and MCL1 isoform effects on apoptosis and metabolism.

The expression of MCL1 is induced through multiple pathways. MAPK1/3 signaling promotes the transcription of MCL1 along with BCL2 and BCL2L1, proteins in the same family that are responsible for the regulation of apoptosis [114]. In melanoma, the relatively high proportion of MCL1 to BCL2L1 highlights its predominant role in preventing apoptosis [115,116]. Furthermore, MAPK1/3 activation also helps retain MCL1 expression by slowing the degradation of the protein via the phosphorylation of threonine 163 [117]. Oncogenic BRAF overexpression, upstream of MAPK1/3, is also associated with higher MCL1 expression [118]. Discordin domain receptor tyrosine kinase 1 (DDR1) has also been implicated in MCL1 expression in uveal melanoma, where the downregulation of DDR1 also decreases MCL1 expression [119]. This pathway is likely mediated by STAT3, as the knockdown of DDR1 led to less STAT3 binding to the promoter region for the MCL1 gene. The inhibition of DDR1 with 7rh, a potential drug compound, to induce apoptosis was resisted by the forced overexpression of MCL1, providing evidence that MCL1 is the component actively resisting apoptosis in DDR1-mediated cell survival [119]. The regulation of MCL1 and the roles of the matrix versus outer mitochondrial membrane isoforms are illustrated in Figure 3.

In melanoma, the high expression of MCL1 protects cancer cells from apoptosis, making it a key target for lowering resistance to treatment [116]. Recent selective MCL1 inhibitors such as AZD5991 have shown synergy with MAPK1/3 inhibitors in inducing apoptosis [31,115]. Thus, combining inhibitors of MCL1 with MAPK1/3 inhibitors has been shown to increase the efficacy of treatments targeting BRAF/MAP2K1 [115,116]. The simultaneous inhibition of MCL1 and BCL2L1 further enhances the induction of apoptosis. In both 2D and 3D spheroid cultures, the combination of BH3-only protein (a class of proteins that inhibit MCL1 and BCL2L1 to induce apoptosis) mimetic drugs targeting MCL1 and BCL2L1 led to a synergetic effect [113,116,120]. For instance, combining MCL1 inhibitor S63845 with BCL2L1 inhibitor A-1331852 led to potent apoptosis at 1 µM concentration each, whereas each drug individually only led to significant effects at 10 µM [120]. Similarly, for non-small cell lung cancer, treatments combining honokiol, a natural compound with antitumor effects, and osimertinimb (Osim), an FDA-approved EGFR inhibitor, display a synergetic effect. The combination with honokiol re-sensitized Osim resistant cells to treatment while inducing apoptosis, likely via the increased degradation of MCL1 [121].

Because of the well-documented role of MCL1 in anti-apoptotic activity, examining its role and regulation through the lens of metabolism could provide more insight on how to target this pro-survival mechanism. The simultaneous inhibition of both glycolysis and oxidative phosphorylation has been shown to decrease MCL1 expression. The reduction in metabolic plasticity via intermittent fasting to limit glucose availability (and therefore glycolysis) and metformin (an inhibitor of oxidative phosphorylation) led to decreases in tumor growth in mice with melanoma xenografts via lowered MCL1 expression [122]. Further examining how limiting metabolic plasticity could lower resistance to apoptosis would provide valuable insight into circumventing mechanisms of treatment resistance in melanoma.

## 5. TRAP1—Stabilizing Mitochondrial Proteins and Inhibiting Oxidative Phosphorylation

Tumor necrosis factor receptor associated protein 1 (TRAP1), an anti-apoptotic mitochondrial protein in the heat shock protein 90 (HSP90) family, is involved in mitochondrial metabolism and known to be altered in cancers. Its regulatory roles in mitochondria include shifting metabolism towards glycolysis, contributing to mitochondrial stability, and combating oxidative stress by lowering ROS [41,123,124,125]. TRAP1 knockout cells displayed increased oxygen consumption, decreased glycolytic metabolites, increased citric acid cycle metabolites, and increased ROS [126,127]. TRAP1 overexpression was associated with mitochondrial fission, while TRAP1 knockdown cells displayed a more fused mitochondrial morphology [127].

TRAP1 also inhibits electron transport chain activity at complex II, succinate dehydrogenase (SDH), and complex IV, cytochrome c oxidase. HIF1A and MYC contribute to transcription of the TRAP1 gene, while the MAPK signaling pathway phosphorylates and subsequently activates TRAP1 [41]. Because of its activity in inhibiting SDH, which catalyzes the conversion of succinate into fumarate, TRAP1 contributes to the stabilization of HIF1A via accumulation of succinate [41,128,129]. Given the role of HIF1A discussed above, TRAP1 stabilization of this master regulator of glycolysis likely contributes to plasticity and aerobic glycolysis. TRAP1 also regulates and opposes the activity of SRC, a factor that drives oxidative phosphorylation [126]. In colorectal cancer cells, TRAP1 has been observed to interact with phosphofructokinase 1, the rate limiting enzyme of glycolysis, to prevent its ubiquitination and degradation, further enhancing glycolytic activity [130].

The levels of TRAP1 in the mitochondria of cancerous cells have been observed to vary widely (both above and below the level of TRAP1 in normal mitochondria) depending on the specific malignancy [126,131]. Because TRAP1 plays a role in shifting metabolism from towards glycolysis, levels of TRAP1 are often elevated in glycolytic cancers, and cancers that more often employ oxidative phosphorylation display lowered TRAP1 levels. These observations make TRAP1 an oncogene or tumor suppressor depending on the metabolic context [126,132].

**Figure 4:**
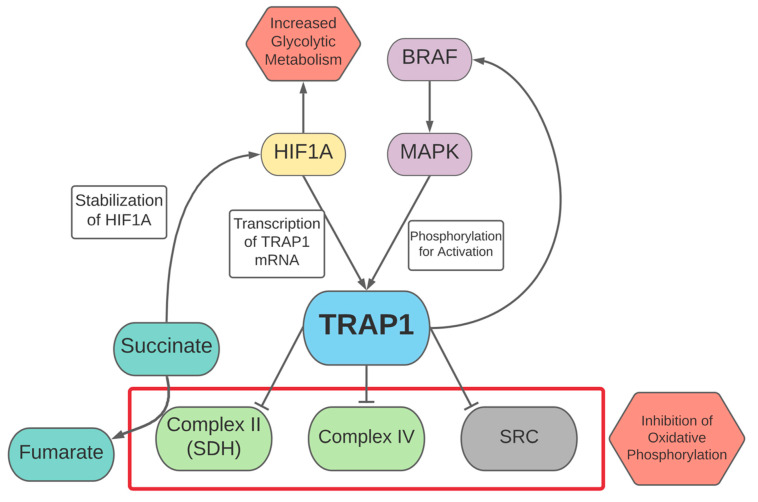
Regulation of TRAP1 and effects of TRAP1 on tumor cell plasticity.

TRAP1 has also been observed to play a role in the regulation of BRAF, subsequently activating the MAPK signaling pathway and inducing the associated metabolic effects described in the BRAF section above [125]. A study on colorectal tumors found a positive correlation between TRAP1 and BRAF levels [42]. Furthermore, the study found that TRAP1/HSP90 inhibitors led to a decrease in BRAF, with this effect appearing more pronounced in colorectal tumor cell lines with the BRAF^V600E^ mutation, suggesting that TRAP1 plays a key role in maintaining BRAF expression [42]. Given the role of BRAF signaling in activating genes associated with glycolysis, this strengthens the role of TRAP1 in modulating a shift towards a more glycolytic metabolism as shown in Figure 4.

Beyond its role in stabilizing a glycolytic metabolism, as a member of the HSP90 chaperones, TRAP1, has been studied as a factor that regulates mitochondrial protein folding and has been implicated in intrinsic resistance to MAPK inhibitors. Because of its importance in maintaining mitochondrial protein folding integrity as a chaperone, TRAP1 inhibition in a study led to decreased oxidative phosphorylation and glycolysis in multiple cancer cell lines, including melanoma cell lines [133]. Although this may seem contradictory to the effect of TRAP1 in inhibiting oxidative phosphorylation, the destabilization of protein folding via the loss of TRAP1 could have a more significant effect on decreasing oxidative phosphorylation than normal TRAP1 inhibitory effects in certain contexts. In melanoma cells, the inhibition of TRAP1 with gamitrnib (a small-molecule mitochondrial HSP90 inhibitor) in conjunction with treatment with MAPK inhibitors showed a synergistic effect, leading to mitochondrial dysfunction and hinderance of tumor bioenergetics and [5,133]. Additionally, overexpression of TRAP1 has been observed to confer resistance to apoptosis in BRAF^V600E^ cells by inhibiting ROS-activated mitochondrial permeability transition pore opening. This effect could contribute to the overall survival of melanoma cells and emphasizes the role of TRAP1 in opposing oxidative stress [41,134].

The role of TRAP1 in plasticity, as shown in its stabilization of a glycolytic metabolism and chaperone function for both glycolytic and respiratory proteins, is significant. Its inhibition may help melanoma move away from a glycolytic metabolism and decrease plasticity overall by destabilizing the proteins needed for glycolysis and oxidative phosphorylation. TRAP1 inhibitors also seem to selectively affect cancer cells and affect normal cells less, making them an attractive option [135]. Treatments that inhibit TRAP1 by interacting with an allosteric pocket, such as honokiol bis-dichloroacetate, have been shown to reverse TRAP1 effects in cancer cells, subsequently decreasing proliferation and tumorigenic growth while restoring ROS levels and SDH function [41,136]. Combining TRAP1 inhibition with chemotherapy has led to synergistic effects in some cancers. For example, the simultaneous use of NVP-HSP990, an inhibitor of HSP90, and melphalan, a chemotherapy drug that alkylates DNA, demonstrated antitumor effects in certain human myeloma cell lines [135]. Given the metabolic plasticity of melanoma, further research can help determine what metabolic profile would be most vulnerable to TRAP1 inhibitory treatment. For example, the overexpression of BRAF and the resulting glycolytic metabolic profile could be targeted by TRAP1 inhibition to possibly reduce plasticity and force cells to adopt a more respiratory profile.

## 6. RHOA and Serum Response Factor (SRF)—Regulating Mitochondrial Fusion versus Fission and Glutaminolysis

Serum response factor (SRF), a transcription factor, has been implicated in resistance to targeted therapies as well as driving metastasis in melanoma [137,138]. RHOA, a member of the Rho family of GTPases, regulates SRF, and this RHOA–SRF signaling axis integrates cytoplasmic events such as actin polymerization from globular actin (G-actin) to fibrous actin (F-actin) [44]. In the context of metabolic activity in melanoma, actin polymerization is key for balancing the fission versus fusion morphology of mitochondria, which in turn influences metabolism [139]. The current state of information suggests that stimuli that allow the polymerization of G-actin to F-actin, such as RHOA activation, release myocardin-related transcription factor A (MRTFA) which is normally associated with G-actin. Consequently, MRTFA is translocated into the nucleus, where it binds as a partner to SRF, allowing transcription of SRF target genes [140]. As a result, polymerized actin interacts directly with mitochondria and induces mitochondrial fission, while depolymerization allows mitochondrial fragments to slowly fuse back together [139].

Well-regulated mitochondrial fusion and fission is key in a range of functions, including metabolism shifts and autophagy, making the fusion/fission dynamic one possible target for potential treatments [141]. Indeed, cancer cells have been reported to have more mitochondria in a fission state compared to normal cells that use oxidative phosphorylation, and the induced fusion of mitochondria reduces cancer cell growth [30]. Specifically in melanoma, one study examining dynamin-1-like protein (DNM1L), which contributes to mitochondrial fission, found disrupted mitochondrial fission via the inhibition of DNM1L-induced melanoma cell death. The same study found that the knockdown of mitofusin 2 (MFN2), key for fusing mitochondria, suppressed the respiratory activity of tumor cells [142]. This seems to suggest that mitochondria in a fission state are more glycolytic compared to their fused counterparts. Further examination of the role mitochondrial fission and fusion plays, and the influence of RHOA signaling on this morphology shift, would contribute to the understanding of melanoma metabolism.

**Figure 5:**
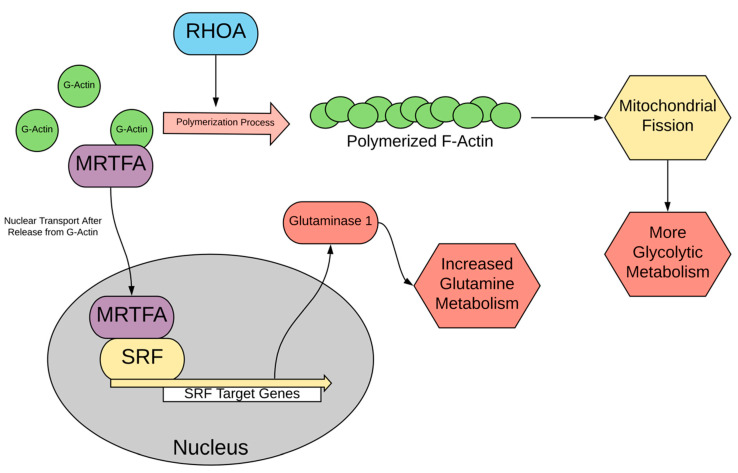
Regulation of RHOA/SRF signaling and effects on metabolism.

Aside from the regulation of fusion/fission, the RHOA–SRF signaling pathway plays a role in regulating a shift towards glutaminolysis in certain melanoma cells, as noted in Figure 5. Targeting this function of RHOA–SRF signaling can induce death in cancer cells that have developed resistance to treatment through the MYC transcription factor [44]. In melanoma, multiple mechanisms of resistance to BRAF and MAP2K1/2 inhibition, including MAPK1/3 reactivation and PI3K-AKT1 pathway activation, converge upon MYC as a key element [143]. MYC, in turn, induces glutamine addiction in cells, as it promotes glutamine uptake and usage as a fuel [144]. A study found that MYC upregulates glutaminase 1 (GLS1) expression to drive glutamine metabolism, and the activation of the RHOA–SRF pathway also upregulates GLS1. Together, MYC and RHOA–SRF synergized to induce GLS1. However, upon the inhibition of RHOA signaling (through C3 transferase, a known RHOA inhibitor), MYC activity in stimulating glutaminolysis was greatly reduced [44]. Furthermore, the activation of SRF partially rescued cells with activated MYC and deactivated RHOA, demonstrating its role as a downstream effector of RHOA. Because of MYC’s dependence on RHOA–SRF activity to metabolize glutamine, the inhibition of the RHOA–SRF pathway appears lethal to cells depending on MYC activity, leaving this vulnerability as a possible treatment target [44].

## 7. SIRT3—Inducing Oxidative Phosphorylation in Melanoma Cells

Sirtuin 3 (SIRT3) is a mitochondria deacetylase which has been proposed to be an oncogene or tumor suppressor, with the predominance of these contrasting roles varying among different types of malignancies [145]. SIRT3 activates multiple catabolic mitochondrial pathways, including activity in metabolizing amino acids, oxidating fatty acids, and promoting oxidative phosphorylation. For instance, it deacetylates NDUFA9 (part of Complex I of the electron transport chain) and SDH (Complex II), thereby increasing electron transport chain activity [32]. Studies have shown increased, decreased, and mutated SIRT3 in a variety of cancers [145]. SIRT3 is induced by PGC1A, and the expression of PGC1A, similarly to the expression of SIRT3, has been both positively and negatively linked to cancer [146,147]. Because aggressive cancers use both respiration and glycolysis, a baseline expression of PGC1A and SIRT3 could help maintain respiration to support tumor growth. Therefore, knockdown of these proteins would reduce mitochondrial plasticity and tumor growth in vivo, and thus SIRT3 would appear to have oncogenic effects. SIRT3 may also have oncogenic activity by regulating ROS levels to prevent apoptosis, thereby enhancing the survivability of cancer cells [148,149]. Conversely, SIRT3 opposes the Warburg effect via the destabilization of HIF1A, preventing cancer proliferation in glycolytic malignancies, such as breast cancer, acting as a tumor suppressor [32,46,148,150]. This effect can be attributed to the role SIRT3 has in controlling ROS and the role of ROS in stabilizing HIF1A activity—with reduced ROS, HIF1A activity is also reduced, preventing the glycolysis and angiogenesis that comes with HIF1A activation [46,151,152].

Furthermore, SIRT3 has a general role in maintaining functional metabolism, as the knockdown of SIRT3 led to alterations in genes for multiple biochemical pathways, including glycolysis, the citric acid cycle, and the pentose phosphate pathway [153]. One key function of SIRT3 is reduction in ROS via the detoxification enzyme manganese superoxide dismutase 2 (SOD2). Increased SOD2, as observed in treatment-resistant melanoma, prevents apoptosis from elevated oxidative stress [26]. Studies of bovine fibroblasts demonstrated that the downregulation of SIRT3 via TGF-β led to altered glucose metabolism and increased ROS under oxidative stress, which could be rescued using hexafluoro to induce SIRT3 expression [47]. Considering the metabolic plasticity observed in melanoma, further examination of the role SIRT3 plays in maintaining the equilibrium of these bioenergetic pathways could lead to a better understanding of how SIRT3 suppression or induction could affect tumors.

Specifically, in melanoma, SIRT3 is upregulated, and knockdown of SIRT3 has led to reduced proliferation. Compared with melanocytic nevi, melanoma tissues show a significant overexpression of SIRT3 [33]. In addition, the experimental overexpression of SIRT3 via plasmids in Hs294T human melanoma cells and immortalized melanocytes led to both enhanced proliferation and colony formation in the melanoma cells and increased proliferation in melanocytes, evidence of the role of SIRT3 in melanoma growth [33]. This result was supported by observations that tumors with an induced overexpression of SIRT3 in mouse xenograft tumors had increased tumorigenicity [153]. Furthermore, short hairpin RNA knockdown of SIRT3 in melanoma led to favorable effects including reduced cell proliferation and migration, indicating possible therapeutic options [33].

While the suppression of SIRT3 counters the growth of melanoma cells, the induction of SIRT3 also could be favorable in certain treatment-resistant melanomas. Examining SIRT3 activators in BRAF inhibitor-resistant melanoma provided a model for how cancers with defective mitochondria, traditionally resistant to current therapies, may be targeted [154]. SIRT3 can be induced by small molecules such as honokiol and hexafluoro, and studies using these small molecule SIRT3 activators have demonstrated antitumor activity in vivo [47]. A study compared a pair of BRAF mutant human melanoma cells: LM36, which is sensitive to vemurafenib, and LM36R, which is vemurafenib resistant. The subsequent induction of SIRT3 with small molecule activators revealed that honokiol DCA and hexafluoro had more activity against the more aggressive and vemurafenib-resistant cell line than the parental cell line [154].

**Figure 6:**
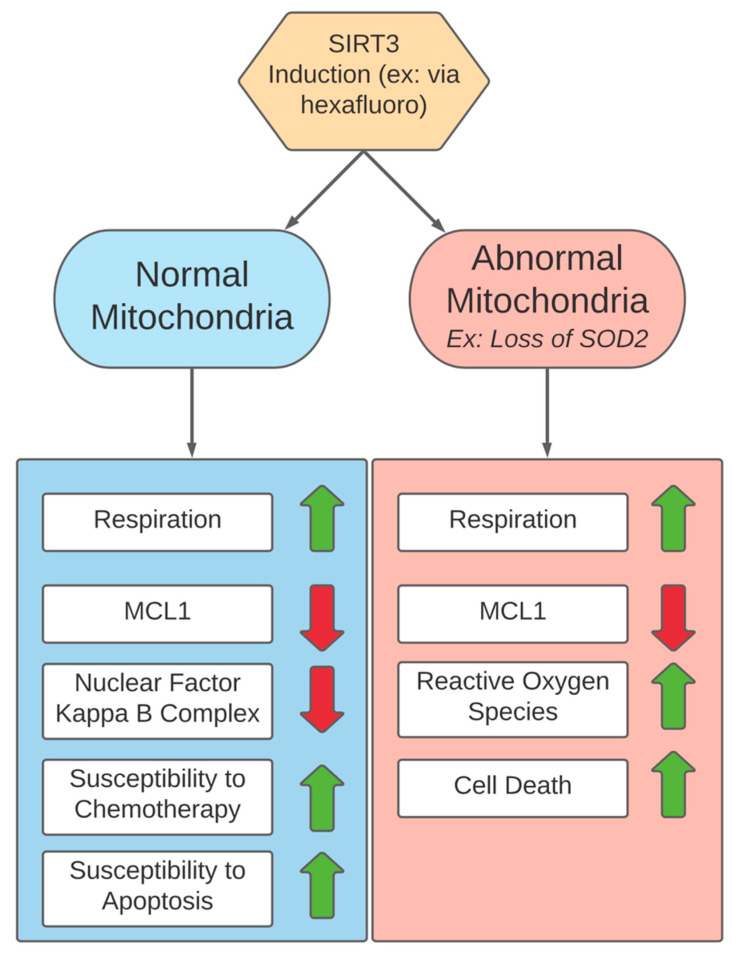
Effects of SIRT3 expression in normal versus abnormal mitochondria.

The analysis of the two cell lines demonstrated that the resistant cell line had lost expression of SOD2, which is associated with a highly aggressive phenotype in melanoma [154]. Interestingly, the SIRT3-driven detoxification of ROS is partially mediated through the activation of SOD2, so this aggressive phenotype with defective detoxification of reactive oxygen could be vulnerable to cytotoxic oxygen in the more oxidative metabolism induced by SIRT3 [32]. The induction of SIRT3 causes the overall amplification of mitochondria, and the amplification of mitochondria that are defective in SOD2 could result in a cytotoxic increase in reactive oxygen [146,154]. Through SIRT3, honokiol derivatives might have driven mitochondrial fusion, a morphology favoring oxidative phosphorylation [154]. Tumors which have high levels of SIRT3 in vivo may have normal or nearly normal mitochondria due to the important regulatory role of SIRT3, while tumors which have lower levels of SIRT3 may have mitochondrial abnormalities and may be susceptible to SIRT3 activation [153]. Furthermore, inducing SIRT3 could reduce MCL1, decreasing resistance to apoptosis, while shifting away from a glycolytic metabolism could decrease chemoresistance mediated by the nuclear factor kappa B complex [18,155]. Effects of SIRT3 induction and the differences in response between normal and abnormal mitochondria are summarized in Figure 6. As melanoma cells can preferentially use a combination of glycolysis and oxidative phosphorylation, examining when to suppress (countering respiration-dependent growth) or induce (targeting weaknesses in ROS detoxification in treatment-resistant melanomas) SIRT3 could be helpful.

Beyond its role in glucose metabolism, SIRT3 has also been noted for its role to control glutamine metabolism and de novo nucleotide biosynthesis in breast cancer. Both cells with silenced (via shRNA) or knocked out SIRT3 displayed increased glutamine uptake and subsequent proliferation [156]. In addition, under conditions of starvation, SIRT3 knockout cells displayed three times the mTORC1 signaling as control cells, showing increased glutamine metabolism and nucleotide biosynthesis [156]. Ultimately, this exposed a vulnerability where treatment with glutamine analogs could reduce proliferation in cells without SIRT3 [156]. This interaction between SIRT3 and glutamine metabolism would be interesting to study in other cancers, such as melanoma, where inhibiting glutamine transport has been considered as a method of suppressing melanoma growth in both wild-type BRAF and BRAF-inhibitor-resistant melanoma [157].

## 8. PTEN and AKT1—Regulating Both Glycolysis and Oxidative Phosphorylation

Phosphatase and tensin homolog (PTEN) mutations can be found in a significant proportion of melanoma cells, with a PTEN mutation rate of 30–50% in melanoma cell lines and 5–20% in uncultured melanoma [48]. Furthermore, the aberrant regulation of AKT1 is also prevalent, found in 43–67% of melanomas [158]. In normal cells, PTEN, a tumor suppressor, dephosphorylates the phospholipid PIP3 and inhibits the AKT1 signaling pathway [79,158,159]. Consequently, mutations in PTEN in melanoma reduce this inhibition and allow for high AKT1 levels, with AKT1 playing a major role in metabolic alterations, resistance to treatment, and melanoma invasiveness [5]. Notably, AKT1 activation is sufficient to transform noninvasive melanoma into an invasive form in vivo [80,160]. Downstream effectors of AKT1, such as the mTORC1 protein, have key effects on the metabolism of melanoma cells [5,55,161].

**Figure 7:**
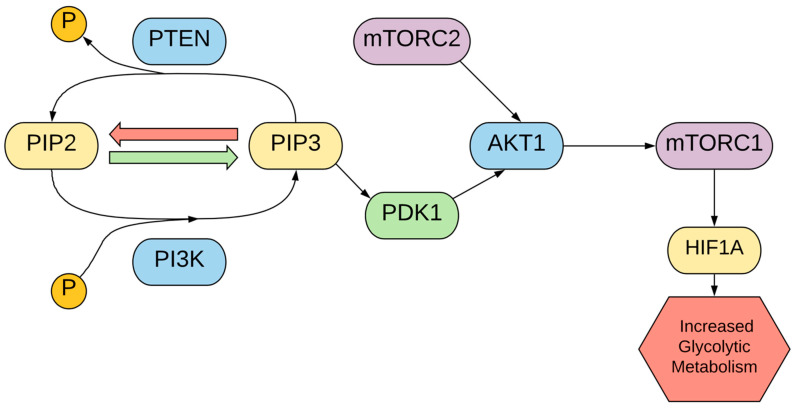
Regulation and effects of PI3K/AKT1/mTORC1 signaling.

Mechanistically, phosphoinositide 3-kinase (PI3K) phosphorylates PIP2, resulting in PIP3, which can be dephosphorylated by PTEN activity. PIP3 recruits AKT1 and PDK1, facilitating the phosphorylation of the threonine 308 residue on AKT1 [162]. AKT1 is also phosphorylated by the mTORC2 protein complex on its serine 473 residue, with these two phosphorylation events activating AKT1 activity [162,163]. AKT1 then activates the protein complex mTORC1, which regulates protein synthesis, cell growth, and cellular proliferation [164]. As cell growth through protein synthesis is important for tumor growth, the PI3K/AKT1/mTORC1 pathway, summarized in Figure 7, is often upregulated in cancers including melanoma [55]. For instance, in other skin cancers such as basal and squamous cell carcinoma, the upregulation of the AKT1 signaling pathway has been implicated in tumorigenesis and hyperproliferation [165]. In addition to protein synthesis, mTORC1 has been proposed as a regulator of HIF1A, contributing to aerobic glycolysis [163,166]. Subsequently, the upregulation of the PI3K/AKT1/mTORC1 axis has been implicated in the upregulation of glycolytic enzymes [5]. Mutations in PTEN, as mentioned above, and the mutation of the PIK3CA gene which leads to the hyperactivity of PI3K contribute to this overactivation.

Evidence suggests that AKT1 upregulates both glycolytic and oxidative activity through multiple mechanisms and allows higher levels of ROS, protecting cells from apoptosis due to oxidative stress [167,168]. In terms of enhancing glycolysis, the effects of AKT1 include the possible indirect activation of phosphofructokinase 1 and increase in HIF1A activity, mediated by mTORC1 as mentioned above, contributing to the Warburg effect [49]. AKT1 also increases coupling efficiency between glycolysis and oxidative phosphorylation via hexokinase association, benefiting kinetics for both processes [50]. In terms of oxidative stress, a study that transfected active AKT1 into WM35 melanoma cells produced an increase in reactive oxygen, particularly superoxide [160]. This ability to generate reactive oxygen while being resistant to oxidative stress could confer a survival advantage, as ROS causes the inactivation of PTEN, p53, and IkB (three important tumor suppressors), preventing the inhibition of AKT1 and allowing the evasion of apoptosis [160]. The activation of AKT1 is also a mechanism of resistance to BRAF inhibition, as AKT1 upregulation was observed to rebound after treatment. PTEN inhibits this increased activity, but subsequent mutations in AKT1, such as AKT1^Q79K^, evaded this inhibition, allowing more effective resistance to treatment [79]. Furthermore, BRAF and MAP2K inhibition-resistant cells show the restoration of mTORC1 activity, regulated by upstream PI3K and AKT1 [169].

Because of the importance of the PI3K/AKT1 signaling axis in cancer proliferation and metabolism, treatments targeting the pathway could be promising. Everolimus, a treatment that inhibits both mTORC1 and mTORC2, has been shown to reduce the invasiveness of melanoma cells [161]. A phase I clinical trial combining the BRAF inhibitor vemurafenib and everolimus showed generally favorable results for patients with advanced cancers, along with no excessive toxicity [170]. In addition, the inhibition of mTOR, a part of both mTORC1 and mTOR2, showed reduced growth in BRAF and MAPK inhibition-resistant cells. The mTOR inhibitors rapamycin and NVP-BEZ235 had favorable effects, inducing apoptosis in resistant cells [169]. However, one consideration is that rapamycin treatment has been shown to increase AKT1 activity through a negative feedback loop through insulin-like growth factor-1 receptor (IGF-1R), so mTOR inhibition may have to be combined with IGF-1R inhibition to prevent AKT1 hyperactivation [171]. A wide range of other synthetic and naturally derived agents have been recorded to inhibit PI3K/AKT1/mTORC1 activity, as reviewed by Chamcheu et al. [172]. Another avenue of treatment targets the relation between PI3K/AKT1 and oxidative stress. It has been postulated that while the elevated ROS levels in melanoma cells provide survival and growth advantages, the over-production of ROS can induce negative effects [55,160]. Because of this, cancer cells could be vulnerable to further oxidative stress beyond a threshold limit. One study found that the induction of ROS levels through Nexrutine raised ROS over a certain threshold in cancer cells, leading to the inhibition of growth by impacting the PI3K/AKT1/mTORC1 signaling pathway [55].

The role of AKT1 in enhancing the capacity of both glycolytic and oxidative metabolism, thereby maintaining bioenergetic levels after treatments such as BRAF inhibition, makes it an important factor to study and potentially target to limit the plasticity of melanoma cells.

## 9. Discussion

Multiple factors contribute to metabolic plasticity in melanoma, with effects that impact glycolysis, oxidative phosphorylation, glutaminolysis, and fatty acid oxidation. As research progresses, more targets are being identified as factors that influence metabolism, with studies on how new treatments may influence metabolism. For instance, a recent study by Abildgaard et al. screened metabolic modulators, which could become potential therapeutic drugs, and their effects on melanoma cells [173]. As our understanding of metabolic plasticity in melanoma evolves, there are a few important considerations to consider. These include the nuance and complexity in metabolic plasticity that highlights the importance of context, additional roles of metabolic factors, and potential side effects of targeted treatments.

Because of the complexity of plasticity, studies sometimes yield contradicting results. For example, the current literature shows some conflicting results regarding the effects of inducing or downregulating TRAP1. One study found that cells overexpressing TRAP1 had enhanced proliferative potential compared to TRAP1 knockdown cells, along with increased tumor metastasis in vivo [127]. However, other studies found that the TRAP1 downregulation of SRC prevents cell invasiveness, as TRAP1 suppression enhanced cell invasiveness, and that TRAP1 levels were inversely related to the expression of genes associated with metastatic potential [126,174]. These seemingly contradictory findings could be the result of differences in the tumor progression context. Just as how different states of melanoma dormancy, metastasis, and recurrence likely have different metabolic profiles, the profile of tumor metabolism likely shifts depending on its tumor microenvironment [5]. TRAP1 induction and subsequent shift towards a glycolytic metabolism may lead to rapid proliferation, especially in early tumor development, where cells could still be vulnerable to oxidative stress. However, in later stages of cancer, increased oxidative stress confers survival advantages including the downregulation of tumor suppressors. In addition, ROS-driven genetic instability could help cells resist treatments or metastasize [41]. Furthermore, a metabolic balance exists in tumor cells, as exemplified by how heightened ROS levels may confer survival and growth advantages in high AKT1 tumor cells, but also makes cells vulnerable to oxidative stress beyond a certain threshold which then inhibits growth [55]. When deciding whether to suppress glycolysis or oxidative phosphorylation in tumors, context is very important, as either metabolic state may confer unique survival and growth advantages depending on the conditions.

In addition, understanding the other roles of major factors that influence metabolism can help direct further research and future treatment. For example, both PTEN and SOX2 play roles in immune evasion. PTEN expression is correlated with the presence of T-cells in the tumor microenvironment, while PTEN negative cells show lowered immune signatures [175]. This immune evasion from reduced tumor-antigen cross-presentation due to PTEN mutations allows melanoma cells to avoid detection by the immune system, contributing to the negative outcome of patients with PTEN loss [175,176]. In addition, SOX2 has been shown to activate the JAK-STAT pathway, which regulates both immune response and tumor progression, while inhibiting SOCS3 and PTPN1, which are regulators of immune signaling. This combination allowed melanoma cells to evade apoptosis by CD8+ T-cells [177]. Given that both PTEN mutations and SOX2 activation had additional roles in resisting immunotherapy, these factors pose additional challenges beyond metabolism. Beyond immune evasion, SIRT3, another factor discussed, showed a role in regulating pluripotency. A study of bovine fibroblasts found that the silencing of Sirt3 led to increases in the expression of POU5F1, SOX2, and NANOG, all factors for pluripotency [178]. These factors are implicated in more treatment-resistant and lethal cancers, with the presence of pluripotency factors correlated with poorer cancer outcomes [179]. When weighing the merits of targeting certain metabolic factors, considering effects outside of metabolism could improve the efficacy of treatment.

Finally, methods of targeting a particular metabolic pathway are important to research. For example, in the MAPK signaling pathway, the inhibition of downstream targets of BRAF might seem appealing, with the prevalence of BRAFV600E mutations and the potential for CRAF to circumvent BRAF inhibition. However, although MAP2K1/2 seems an appealing target with unique allosteric binding sites, the fact that these kinases are in all cells would likely lead to excessive effects on normal cells as well [180]. The selection of appropriate targets is crucial and taking advantage of vulnerabilities unique to cancer cells can help avoid side effects. For example, the amplification of mitochondria via the induction of SIRT3, as mentioned previously, would selectively target aggressive melanomas with the loss of mitochondrial SOD2 mitochondria and induce cell death via ROS [154]. Furthermore, enhancing oxidative stress might disproportionately affect cancer cells that use elevated oxidative stress for growth and survival advantages, as they might more easily be pushed over the threshold of toleration of oxidative stress [55]. Research into targeting treatments that specifically exploit aberrant metabolism that would otherwise confer advantages to cancer cells could be a promising avenue.

## 10. Conclusions

By reviewing pathways through which melanoma cells can alter their metabolism, insights can be gleaned regarding future research and treatment targets. As metabolic plasticity often plays a role in resisting targeted therapies, understanding how the suppression or induction of certain pathways can influence metabolism could improve treatment efficacy. In addition, further research into how metabolism might change in the progression of cancer growth and in response to the tumor microenvironment would better inform treatments targeting metabolism—as the context of metabolic plasticity is key. Combination or sequential therapies targeting metabolism in melanoma can help counter BRAF inhibitor resistance in melanoma, preventing the re-activation of BRAF downstream effectors or countering the shift towards oxidative phosphorylation associated with BRAF inhibition. Furthermore, other methods of targeting important metabolic factors, as reviewed above, could improve future treatments. Reducing metabolic plasticity, driving shifts towards certain metabolic profiles, and exploiting the altered metabolism of melanoma cells might inhibit melanoma or make cells more vulnerable to treatment in certain situations, providing avenues of potential treatment.

## Figures and Tables

**Table 1:** Summary of factors reviewed and respective roles in melanoma plasticity.

Factor	Function	Main Roles in Melanoma Plasticity	Discussed Therapeutic Approaches
BRAF and RAF1 (CRAF)	Protein kinases in MAPK signaling pathway	- Activation of MAPK upregulates HIF1A activity, facilitating a shift to aerobic glycolysis (also known as the Warburg Effect) [5] - Suppresses MITF and PGC1A, factors that drive oxidative phosphorylation [34] - BRAF mutations, particularly BRAF^V600E^ in melanoma, amplify these effects [5] - CRAF activation and other mutations emerge in resistance to treatment with BRAF inhibitors to reactivate MAPK [35]	Vemurafenib Development: FDA-approved for treating BRAF^V600E^ melanoma Mechanism of action: selectively inhibits mutated BRAF^V600E^ kinase [36] Dabrafenib Development: FDA-approved for treating BRAF^V600E^ melanoma Mechanism of action: ATP-competitive binding to BRAF kinase [37] Binimetinib Development: FDA-approved for treating BRAF^V600E^ melanoma in combination with encorafenib, a BRAF inhibitor Mechanism of action: selective MAP2K1/2 inhibitor [38]
SOX2	Transcription factor with multiple roles including maintaining pluripotency	- Enables plasticity in response to acidic extracellular environment, shifting metabolism to one favoring oxidative phosphorylation - Inhibits HIF1A by binding to its promotor [20]	Gefitnib Development: approved by FDA for treating non-small cell lung cancer with EGFR mutations Mechanism of action: selective small-molecule inhibitor of EGFR (a SOX2 upstream activator) [39]
MCL1	Anti-apoptotic protein	- Prevents apoptosis and keeps mitochondria intact - Matrix isoform promotes mitochondrial fusion and ATP production, enhancing oxidative phosphorylation efficiency [31]	AZD5991 Development: phase 1 clinical trial paused (ClinicalTrials.gov identifier: NCT03218683) Mechanism of action: selective MCL1 protein inhibitor, leading to increased apoptosis [40]
TRAP1	Mitochondrial heat shock protein, protein chaperone	- Inhibits complexes II and IV of the electron transport chain and SRC, downregulating oxidative phosphorylation - Stabilizes HIF1A via accumulation of succinate [41] - Activates BRAF signaling pathway [42]	Gamitrinib Development: in phase 1 clinical trial (ClinicalTrials.gov identifier: NCT04827810) Mechanism of action: small-molecule, mitochondrial-targeted HSP90 inhibitor [43] Honokiol bis-dichloroacetate Development: synthesized from the natural compound honokiol Mechanism of action: allosteric inhibitor of TRAP1 [41]
RHOA and SRF	Signaling regulates actin dynamics and other pathways	- Induces polymerization of actin, resulting in subsequent mitochondrial fission [44] - Assists in using glutamine as a source of energy [44]	C3 Transferase Development: bacterial exoenzyme from clostridium botulinum [45] Mechanism of action: irreversible inactivation of RHOA GTP-ase protein [45]
SIRT3	Histone deacetylase protein	- Activates complexes I and II of the electron transport chain - Decreases oxidative stress and reactive oxygen species [32] - Indirectly destabilizes HIF1A [46]	Hexafluoro Development: Analog of natural compound honokiol Mechanism of action: induction of SIRT3 [47]
PTEN	Tumor suppressor that dephosphorylates PIP3	- Acts to maintain normal metabolism via the downregulation of PI3K/AKT1/mTORC1 signaling pathway - Deactivating mutations in PTEN often found in melanoma [48]	Therapies are aimed towards decreasing PI3K/AKT1/mTORC1 signaling, which is the role of PTEN
AKT1	Oncogene and key regulator of cellular growth	- Increased PI3K/AKT1/mTORC1 pathway signaling upregulates HIF1A, contributing to a glycolytic metabolism [49] - Increases coupling of efficiency of oxidative phosphorylation and glycolysis, enhancing bioenergetics in general [50]	Everolimus Development: FDA-approved for treating a variety of malignancies Mechanism of action: binds cyclophilin FKBP-12 which then binds mTOR, inhibiting mTORC1 complex formation [51] Rapamycin Development: natural compound from Streptomyces hygroscopicus, FDA approved for immunosupression Mechanism of action: forms complex with FKBP-12, which allostericaly inhibits mTORC1 [52] NVP-BEZ235 Development: in phase I/II clinical trial (ClinicalTrials.gov identifier: NCT00620594) Mechanism of action: ATP-competitive PI3K and mTOR inhibitor [53] Nexrutine Development: plant extract from Phellodendron trees [54] Mechanism of action: induction of oxidative stress [55]

## Data Availability

Not applicable.
